# Diaqua­(1,4,8,11-tetra­aza­cyclo­tetra­decane-κ^4^ *N* ^1^,*N* ^4^,*N* ^8^,*N* ^11^)copper(II) bis­(2,3,4,5,6-penta­fluoro­benzoate) dihydrate

**DOI:** 10.1107/S1600536810025705

**Published:** 2010-07-07

**Authors:** Nur Syamimi Ahmad Tajidi, Norbani Abdullah, Zainudin Arifin, Kong Wai Tan, Seik Weng Ng

**Affiliations:** a Department of Chemistry, University of Malaya, 50603 Kuala Lumpur, Malaysia

## Abstract

The Cu^II^ atom in the title salt, [Cu(C_10_H_24_N_4_)(H_2_O)_2_](C_6_F_5_CO_2_)_2_·2H_2_O, is chelated by the four N atoms of the 1,4,8,11-tetra­aza­cyclo­tetra­decane (cyclam) ligand and is coordinated by two water mol­ecules in a Jahn–Teller-type tetra­gonally distorted octa­hedral geometry. The Cu^II^ atom lies on a center of inversion. The cations, anions and uncoordinated water mol­ecules are linked by N—H⋯O and O—H⋯O hydrogen bonds, forming a layer structure parallel to (001).

## Related literature

For related (1,4,8,11-tetra­aza­cyclo­tetra­deca­ne)copper carb­oxyl­ates, see: Lindoy *et al.* (2003 ▶); Hunter *et al.* (2005 ▶). 
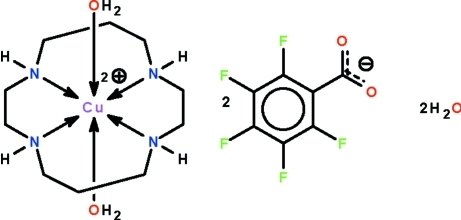


## Experimental

### 

#### Crystal data

[Cu(C_10_H_24_N_4_)(H_2_O)_2_](C_7_F_5_O_2_)_2_·2H_2_O
*M* *_r_* = 758.08
Triclinic, 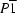

*a* = 7.1976 (6) Å
*b* = 8.7632 (7) Å
*c* = 12.1574 (10) Å
α = 79.378 (1)°
β = 75.408 (1)°
γ = 80.606 (1)°
*V* = 723.85 (10) Å^3^
*Z* = 1
Mo *K*α radiation
μ = 0.87 mm^−1^
*T* = 100 K
0.35 × 0.15 × 0.05 mm

#### Data collection

Bruker SMART APEX diffractometer
Absorption correction: multi-scan (*SADABS*; Sheldrick, 1996 ▶) *T*_min_ = 0.750, *T*_max_ = 0.958
6996 measured reflections
3306 independent reflections
3028 reflections with *I* > 2σ(*I*)
*R*_int_ = 0.026

#### Refinement

*R*[*F* ^2^ > 2σ(*F* ^2^)] = 0.037
*wR*(*F* ^2^) = 0.111
*S* = 1.06
3306 reflections
238 parameters
6 restraints
H atoms treated by a mixture of independent and constrained refinement
Δρ_max_ = 0.49 e Å^−3^
Δρ_min_ = −0.75 e Å^−3^

### 

Data collection: *APEX2* (Bruker, 2009 ▶); cell refinement: *SAINT* (Bruker, 2009 ▶); data reduction: *SAINT*; program(s) used to solve structure: *SHELXS97* (Sheldrick, 2008 ▶); program(s) used to refine structure: *SHELXL97* (Sheldrick, 2008 ▶); molecular graphics: *X-SEED* (Barbour, 2001 ▶); software used to prepare material for publication: *publCIF* (Westrip, 2010 ▶).

## Supplementary Material

Crystal structure: contains datablocks global, I. DOI: 10.1107/S1600536810025705/bt5287sup1.cif

Structure factors: contains datablocks I. DOI: 10.1107/S1600536810025705/bt5287Isup2.hkl

Additional supplementary materials:  crystallographic information; 3D view; checkCIF report

## Figures and Tables

**Table 1:** Hydrogen-bond geometry (Å, °)

*D*—H⋯*A*	*D*—H	H⋯*A*	*D*⋯*A*	*D*—H⋯*A*
N1—H1⋯O2w^i^	0.86 (1)	2.17 (2)	2.997 (2)	157 (3)
N2—H2⋯O1w	0.86 (1)	2.70 (3)	3.123 (2)	112 (2)
O1w—H11⋯O2^i^	0.83 (1)	1.98 (1)	2.785 (2)	162 (3)
O1w—H12⋯O2w	0.83 (1)	2.10 (2)	2.898 (2)	160 (3)
O2w—H21⋯O1	0.83 (1)	1.90 (1)	2.723 (2)	169 (3)
O2w—H22⋯O1^ii^	0.83 (1)	2.08 (2)	2.842 (2)	152 (4)

Symmetry codes: (i) 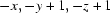; (ii) 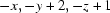.

**Fig. 1.**
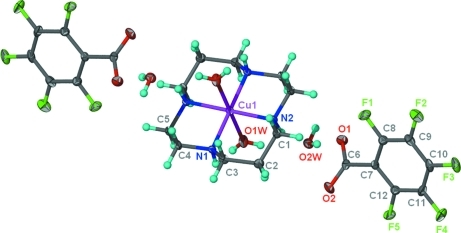
Anisotropic displacement ellipsoid plot (Barbour, 2001) of [Cu(H2O)2(C10H24N4)]2+ 2(C6F5CO2)-.2H2O at the 70% probability level; hydrogen atoms are drawn as spheres of arbitrary radius.

## supplementary crystallographic information

### Comment

The copper(II) ion forms a number of complexes with 1,4,8,11-tetraazacyclotetradecane in which the metal atom is coordinated by the four amino donor-atoms of the cyclic ligand. Among the carboxylate derivatives, neither the acetate nor the benzoate ions bind directly with the copper atom. The copper atom is coordinated instead by water molecules so that the carboxylate group interacts indirectly with the metal atom through the coordinated water molecules (Hunter *et al.*, 2005; Lindoy *et al.*, 2003). The copper(II) atom in the salt, [Cu(H_2_O)_2_(C_10_H_24_N_4_)]^2+^ 2(C_6_F_5_CO_2_)^-.^2H_2_O (Scheme I), is chelated by the four nitrogen atoms of the cyclam ligand and is coordinated by two water molecules in a Jahn-Teller type of tetragonally distorted octahedral geometry. The copper atom lies on a center of inversion (Fig. 1). The cations, anions and lattice water molecules are linked by N–H···O and O–H···O hydrogen bonds to form a layer structure.

### Experimental

1,4,8,11-Tetraazacyclotetradecane (0.50 g, 2.50 mmol) dissolved in ethanol (25 ml) was mixed with a suspension of copper pentafluorobenzoate (1.22 g, 2.5 mmol) in ethanol (50 ml) to give a purple solution. The solution was heated for an hour and then filtered. Prismatic crystals separated from the solution when it was left to cool slowly.

### Refinement

Carbon-bound H-atoms were placed in calculated positions (C—H 0.95 to 0.98 Å) and were included in the refinement in the riding model approximation, with *U*(H) set to 1.2 to 1.5*U*(C).

The water H-atoms were located in a difference Fourier map, and were refined with a distance restraint of O–H 0.84±0.01 Å; their displacement parameters were freely refined.

### Crystal data

**Table:**

[Cu(C_10_H_24_N_4_)(H_2_O)_2_](C_7_F_5_O_2_)_2_·2H_2_O	*Z* = 1
*M**_r_* = 758.08	*F*(000) = 387
Triclinic, *P*1	*D*_x_ = 1.739 Mg m^−^^3^
Hall symbol: -P 1	Mo *K*α radiation, λ = 0.71073 Å
*a* = 7.1976 (6) Å	Cell parameters from 3327 reflections
*b* = 8.7632 (7) Å	θ = 2.4–28.3°
*c* = 12.1574 (10) Å	µ = 0.87 mm^−^^1^
α = 79.378 (1)°	*T* = 100 K
β = 75.408 (1)°	Plate, purple
γ = 80.606 (1)°	0.35 × 0.15 × 0.05 mm
*V* = 723.85 (10) Å^3^	

### Data collection

**Table:**

Bruker SMART APEX diffractometer	3306 independent reflections
Radiation source: fine-focus sealed tube	3028 reflections with *I* > 2σ(*I*)
graphite	*R*_int_ = 0.026
ω scans	θ_max_ = 27.5°, θ_min_ = 1.8°
Absorption correction: multi-scan (*SADABS*; Sheldrick, 1996)	*h* = −9→9
*T*_min_ = 0.750, *T*_max_ = 0.958	*k* = −11→11
6996 measured reflections	*l* = −15→15

### Refinement

**Table:**

Refinement on *F*^2^	Primary atom site location: structure-invariant direct methods
Least-squares matrix: full	Secondary atom site location: difference Fourier map
*R*[*F*^2^ > 2σ(*F*^2^)] = 0.037	Hydrogen site location: inferred from neighbouring sites
*wR*(*F*^2^) = 0.111	H atoms treated by a mixture of independent and constrained refinement
*S* = 1.06	*w* = 1/[σ^2^(*F*_o_^2^) + (0.0585*P*)^2^ + 0.8764*P*] where *P* = (*F*_o_^2^ + 2*F*_c_^2^)/3
3306 reflections	(Δ/σ)_max_ < 0.001
238 parameters	Δρ_max_ = 0.49 e Å^−^^3^
6 restraints	Δρ_min_ = −0.75 e Å^−^^3^

### Special details

**Table:**

Geometry. All e.s.d.'s (except the e.s.d. in the dihedral angle between two l.s. planes) are estimated using the full covariance matrix. The cell e.s.d.'s are taken into account individually in the estimation of e.s.d.'s in distances, angles and torsion angles; correlations between e.s.d.'s in cell parameters are only used when they are defined by crystal symmetry. An approximate (isotropic) treatment of cell e.s.d.'s is used for estimating e.s.d.'s involving l.s. planes.

### Fractional atomic coordinates and isotropic or equivalent isotropic displacement parameters (Å^2^)

**Table:**

	*x*	*y*	*z*	*U*_iso_*/*U*_eq_	
Cu1	0.5000	0.5000	0.5000	0.01239 (13)	
F1	0.30292 (19)	1.10724 (17)	0.18468 (12)	0.0207 (3)	
F2	0.2995 (2)	1.34413 (17)	0.01066 (13)	0.0238 (3)	
F3	−0.0014 (2)	1.41338 (16)	−0.09309 (12)	0.0232 (3)	
F4	−0.3014 (2)	1.23540 (18)	−0.02310 (13)	0.0257 (3)	
F5	−0.29974 (19)	0.99746 (17)	0.14925 (13)	0.0201 (3)	
O1	0.0371 (2)	0.94440 (19)	0.36194 (14)	0.0171 (3)	
O2	−0.0383 (3)	0.78296 (19)	0.26114 (14)	0.0185 (4)	
O1W	0.1535 (2)	0.4892 (2)	0.59710 (15)	0.0184 (4)	
H11	0.095 (4)	0.418 (3)	0.641 (2)	0.027 (8)*	
H12	0.059 (3)	0.558 (3)	0.598 (3)	0.030 (9)*	
O2W	−0.1025 (2)	0.77849 (19)	0.57103 (14)	0.0149 (3)	
H21	−0.057 (4)	0.818 (4)	0.5037 (13)	0.025 (8)*	
H22	−0.101 (5)	0.843 (3)	0.613 (3)	0.038 (10)*	
N1	0.4818 (3)	0.3368 (2)	0.40734 (15)	0.0101 (3)	
H1	0.3625 (19)	0.321 (3)	0.428 (2)	0.018 (7)*	
N2	0.4084 (3)	0.6798 (2)	0.38696 (16)	0.0112 (4)	
H2	0.2840 (15)	0.687 (3)	0.404 (2)	0.015 (7)*	
C1	0.4771 (3)	0.6699 (3)	0.26255 (19)	0.0136 (4)	
H1A	0.6192	0.6702	0.2399	0.016*	
H1B	0.4176	0.7627	0.2179	0.016*	
C2	0.4258 (3)	0.5219 (3)	0.23401 (19)	0.0142 (4)	
H2A	0.2861	0.5155	0.2660	0.017*	
H2B	0.4499	0.5295	0.1494	0.017*	
C3	0.5400 (3)	0.3721 (3)	0.28057 (18)	0.0133 (4)	
H3A	0.5188	0.2837	0.2467	0.016*	
H3B	0.6798	0.3832	0.2572	0.016*	
C4	0.5931 (3)	0.1921 (2)	0.45282 (19)	0.0129 (4)	
H4A	0.7335	0.1972	0.4225	0.016*	
H4B	0.5591	0.1002	0.4289	0.016*	
C5	0.5434 (3)	0.1775 (3)	0.58302 (19)	0.0139 (4)	
H5A	0.4041	0.1679	0.6137	0.017*	
H5B	0.6190	0.0835	0.6163	0.017*	
C6	0.0001 (3)	0.9099 (3)	0.27439 (18)	0.0120 (4)	
C7	0.0021 (3)	1.0433 (2)	0.17373 (18)	0.0109 (4)	
C8	0.1511 (3)	1.1355 (3)	0.13503 (19)	0.0134 (4)	
C9	0.1521 (3)	1.2583 (3)	0.04536 (19)	0.0155 (4)	
C10	−0.0004 (4)	1.2919 (3)	−0.00841 (19)	0.0160 (5)	
C11	−0.1512 (3)	1.2027 (3)	0.0274 (2)	0.0160 (4)	
C12	−0.1483 (3)	1.0797 (3)	0.11656 (19)	0.0133 (4)	

### Atomic displacement parameters (Å^2^)

**Table:**

	*U*^11^	*U*^22^	*U*^33^	*U*^12^	*U*^13^	*U*^23^
Cu1	0.0154 (2)	0.0093 (2)	0.0126 (2)	−0.00158 (14)	−0.00413 (14)	−0.00071 (14)
F1	0.0142 (6)	0.0252 (8)	0.0237 (7)	−0.0079 (6)	−0.0080 (6)	0.0036 (6)
F2	0.0242 (7)	0.0200 (7)	0.0245 (8)	−0.0133 (6)	0.0020 (6)	0.0023 (6)
F3	0.0431 (9)	0.0114 (7)	0.0126 (7)	−0.0011 (6)	−0.0065 (6)	0.0031 (5)
F4	0.0268 (8)	0.0269 (8)	0.0255 (8)	0.0027 (6)	−0.0174 (6)	0.0015 (6)
F5	0.0137 (6)	0.0226 (7)	0.0254 (7)	−0.0070 (5)	−0.0070 (5)	0.0008 (6)
O1	0.0234 (8)	0.0172 (8)	0.0119 (7)	−0.0064 (7)	−0.0057 (6)	0.0003 (6)
O2	0.0269 (9)	0.0103 (8)	0.0185 (8)	−0.0056 (6)	−0.0049 (7)	−0.0001 (6)
O1W	0.0117 (8)	0.0138 (8)	0.0253 (9)	−0.0024 (6)	−0.0008 (7)	0.0039 (7)
O2W	0.0165 (8)	0.0135 (8)	0.0143 (8)	−0.0046 (6)	−0.0025 (6)	−0.0002 (6)
N1	0.0088 (8)	0.0090 (8)	0.0124 (9)	−0.0023 (7)	−0.0021 (7)	−0.0011 (7)
N2	0.0096 (8)	0.0100 (8)	0.0136 (9)	−0.0022 (7)	−0.0027 (7)	−0.0001 (7)
C1	0.0153 (10)	0.0117 (10)	0.0132 (10)	−0.0033 (8)	−0.0043 (8)	0.0024 (8)
C2	0.0164 (10)	0.0154 (11)	0.0112 (10)	−0.0042 (8)	−0.0042 (8)	0.0000 (8)
C3	0.0134 (10)	0.0147 (10)	0.0113 (10)	−0.0029 (8)	−0.0005 (8)	−0.0032 (8)
C4	0.0139 (10)	0.0074 (9)	0.0172 (11)	0.0000 (8)	−0.0041 (8)	−0.0014 (8)
C5	0.0161 (10)	0.0089 (10)	0.0174 (11)	−0.0024 (8)	−0.0068 (8)	0.0009 (8)
C6	0.0097 (9)	0.0120 (10)	0.0121 (10)	0.0006 (8)	−0.0009 (8)	−0.0002 (8)
C7	0.0121 (10)	0.0094 (9)	0.0103 (9)	−0.0005 (8)	−0.0013 (8)	−0.0017 (8)
C8	0.0129 (10)	0.0135 (10)	0.0140 (10)	−0.0016 (8)	−0.0031 (8)	−0.0024 (8)
C9	0.0181 (11)	0.0118 (10)	0.0142 (10)	−0.0054 (8)	0.0027 (8)	−0.0013 (8)
C10	0.0266 (12)	0.0089 (10)	0.0093 (10)	0.0007 (9)	−0.0017 (9)	0.0005 (8)
C11	0.0172 (11)	0.0164 (11)	0.0145 (10)	0.0041 (9)	−0.0074 (8)	−0.0031 (8)
C12	0.0133 (10)	0.0129 (10)	0.0143 (10)	−0.0015 (8)	−0.0030 (8)	−0.0037 (8)

### Geometric parameters (Å, °)

**Table:**

Cu1—N1	2.0149 (18)	C1—C2	1.525 (3)
Cu1—N1^i^	2.0149 (18)	C1—H1A	0.9900
Cu1—N2	2.0313 (18)	C1—H1B	0.9900
Cu1—N2^i^	2.0313 (18)	C2—C3	1.526 (3)
Cu1—O1W	2.4849 (17)	C2—H2A	0.9900
F1—C8	1.345 (3)	C2—H2B	0.9900
F2—C9	1.337 (3)	C3—H3A	0.9900
F3—C10	1.338 (3)	C3—H3B	0.9900
F4—C11	1.340 (3)	C4—C5	1.518 (3)
F5—C12	1.341 (3)	C4—H4A	0.9900
O1—C6	1.258 (3)	C4—H4B	0.9900
O2—C6	1.236 (3)	C5—N2^i^	1.479 (3)
O1W—H11	0.834 (10)	C5—H5A	0.9900
O1W—H12	0.832 (10)	C5—H5B	0.9900
O2W—H21	0.833 (10)	C6—C7	1.528 (3)
O2W—H22	0.830 (10)	C7—C8	1.383 (3)
N1—C3	1.479 (3)	C7—C12	1.393 (3)
N1—C4	1.480 (3)	C8—C9	1.383 (3)
N1—H1	0.859 (10)	C9—C10	1.380 (3)
N2—C5^i^	1.479 (3)	C10—C11	1.376 (3)
N2—C1	1.482 (3)	C11—C12	1.382 (3)
N2—H2	0.861 (10)		
			
N1—Cu1—N1^i^	180.00 (9)	N1—C3—H3A	109.3
N1—Cu1—N2	93.24 (7)	C2—C3—H3A	109.3
N1^i^—Cu1—N2	86.76 (7)	N1—C3—H3B	109.3
N1—Cu1—N2^i^	86.76 (7)	C2—C3—H3B	109.3
N1^i^—Cu1—N2^i^	93.24 (7)	H3A—C3—H3B	107.9
N2—Cu1—N2^i^	180.0	N1—C4—C5	108.02 (17)
N1—Cu1—O1W	88.91 (7)	N1—C4—H4A	110.1
N1^i^—Cu1—O1W	91.09 (7)	C5—C4—H4A	110.1
N2—Cu1—O1W	86.87 (6)	N1—C4—H4B	110.1
N2^i^—Cu1—O1W	93.13 (6)	C5—C4—H4B	110.1
Cu1—O1W—H11	132 (2)	H4A—C4—H4B	108.4
Cu1—O1W—H12	131 (2)	N2^i^—C5—C4	107.48 (17)
H11—O1W—H12	97 (3)	N2^i^—C5—H5A	110.2
H21—O2W—H22	107 (3)	C4—C5—H5A	110.2
C3—N1—C4	111.85 (17)	N2^i^—C5—H5B	110.2
C3—N1—Cu1	118.22 (13)	C4—C5—H5B	110.2
C4—N1—Cu1	105.50 (13)	H5A—C5—H5B	108.5
C3—N1—H1	110 (2)	O2—C6—O1	127.9 (2)
C4—N1—H1	106 (2)	O2—C6—C7	117.14 (19)
Cu1—N1—H1	104 (2)	O1—C6—C7	114.94 (19)
C5^i^—N2—C1	112.36 (17)	C8—C7—C12	116.3 (2)
C5^i^—N2—Cu1	105.50 (13)	C8—C7—C6	122.22 (19)
C1—N2—Cu1	118.01 (14)	C12—C7—C6	121.49 (19)
C5^i^—N2—H2	106 (2)	F1—C8—C9	117.3 (2)
C1—N2—H2	108.4 (19)	F1—C8—C7	120.12 (19)
Cu1—N2—H2	105.8 (19)	C9—C8—C7	122.6 (2)
N2—C1—C2	111.28 (17)	F2—C9—C10	120.0 (2)
N2—C1—H1A	109.4	F2—C9—C8	120.5 (2)
C2—C1—H1A	109.4	C10—C9—C8	119.5 (2)
N2—C1—H1B	109.4	F3—C10—C9	119.3 (2)
C2—C1—H1B	109.4	F3—C10—C11	120.9 (2)
H1A—C1—H1B	108.0	C9—C10—C11	119.7 (2)
C3—C2—C1	113.62 (18)	F4—C11—C10	120.3 (2)
C3—C2—H2A	108.8	F4—C11—C12	120.0 (2)
C1—C2—H2A	108.8	C10—C11—C12	119.7 (2)
C3—C2—H2B	108.8	F5—C12—C11	117.48 (19)
C1—C2—H2B	108.8	F5—C12—C7	120.30 (19)
H2A—C2—H2B	107.7	C11—C12—C7	122.2 (2)
N1—C3—C2	111.72 (18)		
			
N2—Cu1—N1—C3	38.53 (15)	C12—C7—C8—F1	−179.30 (19)
N2^i^—Cu1—N1—C3	−141.47 (15)	C6—C7—C8—F1	1.4 (3)
O1W—Cu1—N1—C3	125.34 (15)	C12—C7—C8—C9	0.5 (3)
N2—Cu1—N1—C4	164.50 (13)	C6—C7—C8—C9	−178.8 (2)
N2^i^—Cu1—N1—C4	−15.50 (13)	F1—C8—C9—F2	0.0 (3)
O1W—Cu1—N1—C4	−108.70 (13)	C7—C8—C9—F2	−179.9 (2)
N1—Cu1—N2—C5^i^	−165.17 (14)	F1—C8—C9—C10	180.0 (2)
N1^i^—Cu1—N2—C5^i^	14.83 (14)	C7—C8—C9—C10	0.1 (4)
O1W—Cu1—N2—C5^i^	106.10 (14)	F2—C9—C10—F3	−1.8 (3)
N1—Cu1—N2—C1	−38.68 (15)	C8—C9—C10—F3	178.2 (2)
N1^i^—Cu1—N2—C1	141.32 (15)	F2—C9—C10—C11	179.7 (2)
O1W—Cu1—N2—C1	−127.41 (15)	C8—C9—C10—C11	−0.3 (3)
C5^i^—N2—C1—C2	179.95 (17)	F3—C10—C11—F4	0.7 (3)
Cu1—N2—C1—C2	56.8 (2)	C9—C10—C11—F4	179.1 (2)
N2—C1—C2—C3	−69.8 (2)	F3—C10—C11—C12	−178.7 (2)
C4—N1—C3—C2	−179.63 (17)	C9—C10—C11—C12	−0.3 (3)
Cu1—N1—C3—C2	−56.8 (2)	F4—C11—C12—F5	−0.2 (3)
C1—C2—C3—N1	69.8 (2)	C10—C11—C12—F5	179.2 (2)
C3—N1—C4—C5	172.46 (17)	F4—C11—C12—C7	−178.4 (2)
Cu1—N1—C4—C5	42.67 (18)	C10—C11—C12—C7	1.0 (4)
N1—C4—C5—N2^i^	−58.0 (2)	C8—C7—C12—F5	−179.24 (19)
O2—C6—C7—C8	−132.7 (2)	C6—C7—C12—F5	0.1 (3)
O1—C6—C7—C8	47.8 (3)	C8—C7—C12—C11	−1.1 (3)
O2—C6—C7—C12	48.0 (3)	C6—C7—C12—C11	178.2 (2)
O1—C6—C7—C12	−131.5 (2)		

Symmetry codes: (i) −*x*+1, −*y*+1, −*z*+1.

### Hydrogen-bond geometry (Å, °)

**Table:**

*D*—H···*A*	*D*—H	H···*A*	*D*···*A*	*D*—H···*A*
N1—H1···O2w^ii^	0.86 (1)	2.17 (2)	2.997 (2)	157 (3)
N2—H2···O1w	0.86 (1)	2.70 (3)	3.123 (2)	112 (2)
O1w—H11···O2^ii^	0.83 (1)	1.98 (1)	2.785 (2)	162 (3)
O1w—H12···O2w	0.83 (1)	2.10 (2)	2.898 (2)	160 (3)
O2w—H21···O1	0.83 (1)	1.90 (1)	2.723 (2)	169 (3)
O2w—H22···O1^iii^	0.83 (1)	2.08 (2)	2.842 (2)	152 (4)

Symmetry codes: (ii) −*x*, −*y*+1, −*z*+1; (iii) −*x*, −*y*+2, −*z*+1.
